# Supplementary material for: Chromothripsis during telomere crisis is independent of NHEJ, and consistent with a replicative origin
Source: Genome Res. 2019 May;29(5):737–49. doi: 10.1101/gr.240705.118 (PMC6499312; doi:10.1101/gr.240705.118)
Supplement: Supplemental Material [file supp_gr.240705.118_Supplemental_file_1.zip › contigs/annotated_contigs/DB45/contig.2.DB45_length_484_mean_cov_4.02892561983.docx]

**DB45_length_484_mean_cov_4.02892561983**

```
TTTCTTATCATCTCAATCTTCTCAACTCTGTAAGATTGGCACATTTCTTTCCCATGTTATGAAGGACAGAACTAAGGTAGAGTAGTTAA
 >chr11:94215363-94215568 + E=8e-112
GTGACTAACATAATTTTACAACTACTAAATGACAGAACCAGGATTTGAACTCCAGCTGTTTTATACCGGAACCCATGCTCTTAATCACT

ATGCTAAACTTTCTAGCAACTCACCTA|TATATATGC|ACACAGGGTACACTCGTCCTCCTGCAGCTTTGGGAGGAAGAGAGTGGGGGC
                                     >chr22:39823806-39824052 - E=2e-118
CTCAGGCAGCAGCTGAGGAGAAACCCAGGGTAGCAGGGGCATAGCTGCTCCATGGCCTGATGGGGTGGTGCCTGAGGGCTGAGTGTGGG

TGGCGGCCATGCCTGGCTCCCCAAGCCCAGTGGGGCCCCCTGCCCCTGGGAACAACTCAGGAGGAGTTTCCCTTGTCAGATACTCTTCC

AGGCCGGGCTGCGAACT|GCTTAATGCTGTAAAGCCTACTGG
```
